# Mapping evidence on access to healthcare information by women of reproductive age in low-and-middle-income countries: scoping review protocol

**DOI:** 10.1186/s13643-019-1203-5

**Published:** 2019-12-16

**Authors:** Joyce T. Shatilwe, Tivani P. Mashamba-Thompson

**Affiliations:** Displine of Public Health Medicine and School of Nursing, University of KwaZulu-Natal, Durban, South Africa

**Keywords:** Access, Health care information, Women of reproductive age, Low- and middle-income countries

## Abstract

**Background:**

Research shows that there are inadequate interventions in resource-limited settings that could enable women of reproductive age to access and use health services in those settings. The main objective of this scoping review is to map the evidence on access to healthcare information by women of reproductive age in LMICs.

**Method and analysis:**

The primary search will include Google Scholar, Science Direct, PubMed, EBSCOhost (Academic search complete, CINAHL with full text, MEDLINE with full text, MEDLINE), Emerald, Embase, CDSR, PsycINFO, published and peer review journals, organisational projects, conference papers, reference list, grey literature sources, as well as reports related to this objective will be included in the study. Identified keywords will be used to search articles from the studies. The articles and abstracts will be screened by two independent reviewers (JS and TPMT). Inclusion and exclusion criteria will be considered to guide the screening. A thematic content analysis will be used to present the narrative account of the reviews, using NVivo computer software (version 11).

**Discussions:**

The scoping review will focus on women of reproductive age in LMICs. We anticipate finding relevant literature on the interventions aimed at accessing health care services in LMICs. The study findings will help reveal research gaps to guide future research.

**Scoping review registration:**

Not registered with PROSPERO (not needed).

**Protocol and registration:**

This scoping review was not registered.

## Background

There is a global commitment to achieve universal health coverage (UHC). Universal health coverage is defined as ensuring that all people have access to needed health services (including prevention, promotion, treatment, rehabilitation and palliation) of sufficient quality to be effective while also ensuring that the use of these services does not expose the user the financial hardship [1, 2]. People in poor countries tend to have less access to health services than those in better-off countries, and within countries, the poor have less access to health services. Whereas the poor in LMICs are consistently at a disadvantage in each of the dimensions of access and their determinants, this need not be the case [3, 4].

The lifetime risk of death for women in high-income countries, where health services are more accessible is 1 in 2400, but in low-income countries, it is 1 in 180 and under conditions of fragility and conflict, it is even high with 1 in every 54 women dying due to complications related to pregnancy [5]. Adolescent fertility is also high in countries with high fertility. More than a fourth of girls and women in sub-Saharan Africa cannot access family planning services, fuelling unplanned pregnancies and maternal, infant and child mortality and morbidity. Adolescent girls are more likely to experience complications due to pregnancy such as obstructed labour and eclampsia increasing their risk of death. Children born to adolescents are also more likely to have a low birth weight, ill health, stunting and other poor nutritional outcomes [5]. Access to health care is a major public health challenge, especially in developing countries [6, 7]. The meagre resources in low-income countries limit access to health services than high-income countries. So is true for sub-Saharan Africa where accessibility of health care services still plays a hurdle for optimum health system performance [6, 8]. Nearly all (99%) maternal deaths occur in developing countries, reflecting inequalities in access to quality health services, these inequalities are reflected in the disparities between high- and low-income women and those living in rural versus urban areas in low- and middle-income countries [9].

A key function of health systems is implementing interventions to improve health, but coverage of essential health interventions remains low in low-income countries. Implementing interventions can be challenging particularly if it entails complex changes in clinical routines, in collaborative patterns among different health care and disciplines, in the behaviour of providers, patients or other stakeholders or in the organisation of care [10]. Barriers to access health care have been widely studied; however, effective interventions to overcome barriers and increase access by women of reproductive age to health care information are less well documented [11].

The Sustainable Development Goal (SDG) 3 which is focused on good health and well-being is aiming to ensure healthy lives and promote well-being for all ages [12]. The inclusion of UHC in the SDGs presents an opportunity to promote a comprehensive and coherent approach to health, focussing on health systems strengthening (HSS) [7]. Many different approaches are shown to improve access to the poor, using targeted or universal approaches, engaging government, non-governmental or commercial organisations and pursuing a wide variety of strategies to finance and organize services [7, 9]. UHC cuts across all health targets and contributes to health security and equity [2, 13, 14]. Apart from UHC, mHealth has the potential to reduce inequalities in care through a variety of applications that aim to facilitate communication between clients and providers, promote women’s behavioural change [9].

A scoping review of the literature regarding the evidence on access to healthcare information by women of reproductive age in low- and middle-income countries is to be conducted. Evidence on interventions which are currently used by different countries in low- and middle-income countries is needed to benchmark and to recommend similar interventions to those countries who are not practising them. The main objectives of this literature are to map the literature on access to healthcare information by women of reproductive age in low- and middle-income countries. The second reason for conducting this specific scoping review is to examine broad areas to identify gaps on access to healthcare information by women of reproductive age in low- and middle-income countries, clarify key concepts and report on the types of evidence that address and inform practice on this topic area (access to healthcare information by women of reproductive age in low- and middle-income countries). A third reason, it is to provide a map of the range of available evidence that can be undertaken as a preliminary exercise prior to the conduct of a systematic review [15, 16].

## Aims/objectives

The main objective of this literature is to map the evidence on access to healthcare information by women of reproductive age in Low-and-Middle-Income Countries.

## Methods

This study is part of a larger study aimed at assessing the barriers and challenges to access and utilise maternal healthcare information provided to young women during pregnancy in the Ohangwena Region, Namibia. We will conduct a scoping review, which will be guided by PRISMA–ScR (Preferred Reporting Items for Systematic review and Meta-Analyses extension for Scoping Review). The PRISMA-ScR follows the following steps: protocol and registration, eligibility criteria, information sources, search, selection of sources of evidence, data charting process, data items, critical appraisal of individual sources of evidence and synthesis of results. Results will include the following steps: Selection of sources of evidence, characteristics of sources of evidence, results of individual sources of evidence and synthesis of results. The last components will be Discussion which involves summary of evidence. Limitations and Conclusion are the last two steps of this methodology [17].

A scoping review requires a broad question to help you investigate what has been done in the field [18]. The type of systematic review will be guided by the study designs of the eligible studies following the screening for the scoping review [19, 20]. A search strategy will be developed with assistance from the librarian. This scoping review will include all study designs. It will also include grey literature. The aim of the study is to map existing evidence to help us answer our research question.

1. Identify the research question

Main research question: What is the evidence on access to healthcare information by women of reproductive age in low- and middle-income countries?

Sub-questions: What factors contribute to women of reproductive age in LMICs to not access health care information?

## Eligibility criteria

Studies will be selected according to the “population-concept-context (PCC)” framework recommended by the Joanna Briggs Institute for scoping reviews as stipulated under Table 1 [19, 21].

**Table 1:** PCC framework

	Criteria	Determinants
P	Population	• Women of reproductive age • Age 14–49
C	Concept	• All study designs with relevant interventions such as: - Any interventions that enable women of reproductive age access to health care information carried out during 2014 to recent.
C	Context	• Research articles will be limited to lower- and middle-income countries. • No restriction on language • Evidence from the period 2004–until recent

The lower- and middle-income countries under the context will be determined by the World Bank list of economies [22] which has classified the countries according to their economic status. The study will focus on the period from 2004–2019, focusing on a 15-year period will enable the researchers to collect broader knowledge from studies generated during the specified period. The study will include articles written in all languages and will use the University of Kwazulu-Natal Systematic Review Services for help with searching for interpreters in case there are studies retrieved that are published in other languages. According to WHO, women of reproductive ages range from 14–49 years old [23].

## Information sources

A suitable team with content and methodological expertise will be assembled to ensure successful completion of the study. A literature search will be conducted from the following databases: Scholar, Science Direct, PubMed, EBSCOhost (Academic search complete, CINAHL with full text, MEDLINE with full text, MEDLINE and PsycINFO), Emerald, Embase and Cochrane Database of Systematic Review (CDSR) published and peer-reviewed journals, organisational projects, conference Google papers, reference list, grey literature, as well as reports related to this objective will be included in the study [19, 24].

Researchers will keep record of the number of publications retrieved and the search dare after each session. Databases will be searched for the data on evidence on access to healthcare information by women of reproductive age in low- and middle-income countries The following keywords will be used: Access, Health care information, Women of reproductive age, Low and Middle Income Countries.

## Selection of sources of evidence

The researcher will search the articles’ titles using keywords search in the following databases: Google Scholar, Science Direct, PubMed, EBSCOhost (Academic search complete, CINAHL with full text, MEDLINE with full text, MEDLINE), Emerald, published and peer-reviewed journals, organisational projects, conference papers, reference list, grey literature, as well as reports related to this objective will be included in the study [19, 24].

This study will be conducted using a three-stage mapping strategy. The first stage will include comprehensive title screening which will include comprehensive title screening which will be done by one reviewer. All eligible studies will be uploaded onto Covidence computer software and duplicates will be removed. The final Covidence library will be shared among the review team. The second and third stage which will comprise of an abstract and full article screening, respectively, will be conducted by two independent reviewers (J.S and TPMT) guided by the inclusion and exclusion criteria. A form with questions will be developed to screen the abstract based on the eligibility criteria [25]. Covidence computer software will be used to upload the selected articles which are relevant to the study. Discrepancies between reviewers will be solved through consensus and involving a third reviewer. Search results from different electronic databases will be combined in a single Covidence library [18, 24].

Inclusion criteria:

To be included, studies must meet the following criteria:

- Study will include women of reproductive age
- Women ranging between the ages of 14–49 years
- Evidence from the period 2004 to recent
- Study contacted in lower- and middle-income countries
- All study designs with relevant interventions
- Studies that focus on interventions enabling access to health services
- Grey literature resources include government and non-governmental organisation reports and academic dissertations
- All studies will be included irrespective of their study designs

Exclusion criteria:

Publications reporting on the following will be excluded:

- Studies that do not focus on interventions aimed at enabling access to health services
- Studies that do not focus on health services
- Women below 13 years old and above 49 years old will not be included in the study
- Study done earlier than 2004
- Studies reporting on non-health interventions

## Data charting process

The research team will collectively develop the data charting form and determine which variables to extract in order to answer the research question. Furthermore, the form will be piloted by two independent reviewers (JS and TPMT). A random sample of 10 included studies will be used for consistency. Based on the feedback from the two reviewers, the data extraction form will be modified as required. Data charting form are being developed and variables and themes to be extracted will be determined in order to answer the research objective [15, 19, 24]. The researcher will continually extract data and update the data-charting form and meet to determine whether their approach to data extraction is consistent with the research question and purpose [19, 26, 27].

## Data items

The data charting form will include a mixture of general information about the study and specific information related to the study population, the type of intervention, outcome measures employed and the study design. The information will include the following: author(s), year of publication, journal full reference, aims or research questions, participant characteristics, recruitment context, sampling method, study design, theoretical background, data collection method, data analysis, intervention, intervention outcome, most relevant findings, conclusion and comments [28]. The NVivo computer software (version 15) shall be used to classify, sort, arrange and examine relationships in the data and to extract the relevant outcomes and for the thematic analysis of the studies.

## Critical appraisal of individual sources of evidence

The quality of the evidence obtained from the studies will be assessed to make sure that the study design is appropriate for the research objectives, to eliminate risk of bias and that the studies are reported well [19]. We will include quality appraisal step to assess the quality of the included studies, as recommended by Levac et al. [15]. The quality of the included studies will be assessed by use of the Mixed Method Appraisal Tool (MMAT) Version 2018 [29]. The Mixed Method Appraisal tool (MMAT) is a checklist that was developed to provide a quality appraisal tool for quantitative, qualitative and mixed methods studies included in systematic mixed studies reviews [30]. Mixed studies reviews synthesise quantitative, qualitative and mixed methods studies that relate to a particular question [29]. The Mixed Method Appraisal Tool (MMAT) provides a set of criteria for concomitantly appraising the methodological quality of quantitative, qualitative and mixed methods studies included in a mixed studies review [29]. The MMAT allows the appraisal of most common types of study methodology and design. This tool will enable us to examine the quality of the included studies such as the appropriateness of the aim of the study, adequacy and methodology, study design, participant recruitment, data collection, data analysis, presentation of findings, authors’ discussions and conclusions [18]. An overall quality score can be ascertained using this tool for each included study [29].

## Synthesis of results

The narrative account will be produced in two ways: First, attention will be given to basic numerical analysis of the extent, nature and distribution of the studies included in the review. Tables and charts mapping will be produced. This will include the distribution of studies geographically and for the different care recipient groups, the range of interventions included in the review, the research methods adopted and the measures of effectiveness used [18, 19, 24, 29]. This part of the analysis will shed light on the dominant areas of research in terms of intervention type, research methods and geographical location.

Secondly, the literature will be organised thematically, according to the two interventions (increased access to health services and health technology) mentioned above. This stage will be divided into 3 steps: (1) Analysis (including descriptive numerical summary analysis and qualitative thematic synthesis) will be conducted. (2) The results will be reported and the outcome will be produced that will refer to the overall purpose or research question. (3) The meaning of the findings will be considered as they relate to the overall study purpose; the implications for future research, practice and policy will be discussed [26].

A synthesis plan will be clearly outlined. A narrative summary will be provided. For collating and summarising results, a consistent approach to reporting the findings will be implemented. The researchers will conduct a content thematic analysis on included studies [31] and present emerging themes in line with the research questions of the study using NVivo computer software (version 15).

Screening results will be reported using the adapted Preferred Reporting Items for Systematic Review and Meta-analysis guidelines (PRIMA) (Additional files 1 and 2) [32]. A template will be developed that will be applied to each intervention group. The template will begin with a small table summarising basic characteristics of all the studies included in that particular interventions group. It will be then followed by commentary written under the following nine headings [27]: interventions, sample sizes, participants, research methods, outcomes, evidence relating to effectiveness, economic aspects, countries studies and gaps in the research [15, 18, 19, 30].

Comparisons will be made across the intervention types and contradictory evidence regarding specific interventions and gaps in the evidence about individual intervention and across interventions as well as consider possible new frontiers will be identified [15, 18, 28, 34].

The identification of research gaps in our study relied on a source such as the literature review, which will be confined to identifying areas of overall weakness within the field by comparing intervention types and study designs [28].

The processes below will be followed [33, 34]:

- Coding data from the included articles
- Categorising the coding into major themes
- Build theme-related themes (cut-and-paste technique)
- Displaying the data
- Identifying key patterns in the data and identify sub-themes
- Summarising

## Discussion

The aim of the study is to map the literature that indicates the evidence on interventions aimed at enabling access to health care information in LMICs. This study preceded the larger study aimed at assessing the barrier and challenges to access and utilise maternal healthcare information in one of the region in Namibia. Getting prior information on the available studies conducted on the topic in LMICs will strengthen the necessity of conducting the main study. It will also enlighten new researchers to conduct more research study on the topic if it found that less study was conducted in return that new knowledge will be generated. This is in support of the Sustainable Development Goal (SDG) 3 which is focused on good health and well-being aiming at ensuring the healthy lives and promotes the well-being of all ages [12]. Sustainable Development Goal (SDG) 3 is also aiming to reduce some of the common killers associated with child and maternal mortality. It is also in support of the UHC in the SDGs which presents an opportunity to promote a comprehensive and coherent approach to health, focussing on health systems’ strengthening (HSS). UHC cuts across all health targets and contributes to health security and equity [2]. It is crucial to have up-to-date information because of daily new developments. Due to a high number of maternal mortality facing countries, it is necessary to follow the latest development since this topic is high on the agenda of the Namibian President’s plan and this study will inform the country’s agenda. Based on this reason, this study will focus only on evidence that has been published from the past 5 years, 2014–2018.

We anticipate finding relevant literature on the distribution of interventions aimed at enabling access to health care information in LMICs. The study findings will help pave way for the systematic review and it will reveal research gaps to guide future research. At the same time, the outcome of the study will be used to determine the type of systematic review to be conducted. Other scholars who are going to do scoping review in this area of study will also benefit by using the outcome of the study. Planners and policy makers in LMICs may find these findings useful during planning and decision-making.

## Supplementary information

**Additional file 1:** PRISMA-P Checklist.

**Additional file 2:** Result of Pilot search in journals.

## Data Availability

All data generated or analysed during this study will be included in the published systematic review article.

## Abbreviations

PRISMA: Preferred Reporting Items for Systematic Review and Meta-Analysis
UNFPA: United Nations Population Fund
SDG: Sustainable Development Goal
AFHS: Adolescent friendly health services
MHC: Maternal and child health
NCDs: Non-communicable diseases
LMICs: Lower- and middle-income countries
PICOS: Population, Intervention, Comparison, Outcomes and Study setting
STATA: Statistical analysis software
BREC: Bio-medical research committee
PCC: Participants-concept-context
UKZN: University of KwaZulu-Natal
UHC: Universal health coverage
