# Supplementary material for: Mapping evidence on access to healthcare information by women of reproductive age in low-and-middle-income countries: scoping review protocol
Source: Syst Rev. 2019 Dec 16;8:328. doi: 10.1186/s13643-019-1203-5 (PMC6913006; doi:10.1186/s13643-019-1203-5)
Supplement: Supplementary file 1 — Additional file 1: PRISMA-P Checklist. [file 13643_2019_1203_MOESM1_ESM.docx]

**Figure 1: PRISMA 2015 flow chart diagram which shows phases of the literature search for extraction of the most specific literature for the review.**
