# Supplementary material for: Mapping evidence on access to healthcare information by women of reproductive age in low-and-middle-income countries: scoping review protocol
Source: Syst Rev. 2019 Dec 16;8:328. doi: 10.1186/s13643-019-1203-5 (PMC6913006; doi:10.1186/s13643-019-1203-5)
Supplement: Supplementary file 2 — Additional file 2: Result of Pilot search in journals. [file 13643_2019_1203_MOESM2_ESM.docx]

**Table 2:** Results of pilot search in PubMed.

| **Search Date** | **Search Engine** | **Keyword search** | **Number of publications retrieved** | **Search terms** |
| --- | --- | --- | --- | --- |
| 11/05/2019 | PubMed | Acess, Health care information, women of reproductive age, Low and Middle Income Countries. | **16136** | **((Access[All Fields] AND (("women"[MeSH Terms] OR "women"[All Fields]) AND ("reproduction"[MeSH Terms] OR "reproduction"[All Fields] OR "reproductive"[All Fields]) AND ("Age"[Journal] OR "Age (Omaha)"[Journal] OR "Age (Dordr)"[Journal] OR "Adv Genet Eng"[Journal] OR "age"[All Fields]))) AND (("delivery of health care"[MeSH Terms] OR ("delivery"[All Fields] AND "health"[All Fields] AND "care"[All Fields]) OR "delivery of health care"[All Fields] OR ("health"[All Fields] AND "care"[All Fields]) OR "health care"[All Fields]) AND ("Information (Basel)"[Journal] OR "information"[All Fields]))) OR (Low[All Fields] AND Middle[All Fields] AND ("income"[MeSH Terms] OR "income"[All Fields]) AND Countries[All Fields])** |
